# Dual homologous recombinase‐mediated lineage tracing technique: New insights for lung stem cell repair

**DOI:** 10.1002/mco2.733

**Published:** 2024-09-21

**Authors:** Yujiao Wu, Zhenwei Xia, Min Wu

**Affiliations:** ^1^ Department of Pediatrics Ruijin Hospital affiliated to Shanghai Jiao Tong University School of Medicine Shanghai China; ^2^ Wenzhou Traditional Chinese Medicine Hospital of Zhejiang Chinese Medical University Wenzhou China; ^3^ Wenzhou Institute University of Chinese Academy of Sciences Wenzhou China

**Keywords:** alveolar epithelia, club cells, lineage tracing, lung regeneration, stem cells

1

In a recent study published in *Cell*, Liu et al. developed a dual homologous recombinase‐mediated genetic profiling tracer technique to accurately track lung epithelial cells, which reveals the regenerative origin and regulatory mechanism of adult alveolar epithelial stem cells, offering new techniques for lung stem cell repair and regeneration.^1^

The ability of adult stem cells to maintain tissue homeostasis and facilitate post‐injury healing is essential. Alveolar regeneration is essential for preserving the integrity of the alveolar epithelial barrier and an adequate gas exchange surface in homeostasis and lung damage repair. Although lung tissue has a modest steady‐state cell turnover, it contains stem cells that are unique to a given location, which rapidly mobilizes in response to tissue damage to regenerate the epithelium. Alveolar type II (AT2) cells serve as alveolar stem cells that will proliferate and differentiate into new alveolar type Ⅰ (AT1) cells that are specialized for gas exchange in the alveoli, maintaining lung homeostasis and facilitating regeneration following injury.^2^ A repair imbalance of this kind can lead to potentially deadly lung diseases. How alveolar stem cells are replenished to preserve this healing equilibrium is currently unclear, though. One effective way to investigate stem cell lineage and cell fate determination is by genetic lineage tracing. The most popular method for tracking stem or progenitor cell lineages in vivo is the Cre‐loxP recombination system. The specificity of Cre expression in the targeted stem or progenitor cells determines how precise this genetic system is. However, Cre expression in non‐targeted cell types has generated debate in numerous prior studies and can make lineage‐tracing studies more difficult to understand.^3^ Recent research suggests that AT2 cells may originate from AT1 and club cells post‐lung damage, but controversy surrounds their regenerative origin due to non‐specific labeling issues using conventional lineage tracing techniques.^4^ In the present study, the authors developed a new dual homologous recombinant lineage tracer based on Cre‐loxP and Dre‐rox, which is capable of accurately labeling alveolar stem cells. This novel method makes it possible to analyze cell lineage and destiny decisions more precisely by overcoming the technical challenge of non‐specific Cre expression. This makes it easier to research stem and progenitor cell plasticity in illness and regeneration in vivo.

The researchers clarified that AT1 cells are terminally differentiated cells that lack plasticity and do not differentiate into AT2 cells following lung injury. The authors discovered that both club cells and bronchioalveolar stem cells (BASCs) could differentiate into the alveolar epithelium and promote alveolar regeneration after lung injury by creating a new dual homologous recombination tool based on Cre‐loxP and Dre‐rox (Figure 1A). These findings confirm the results of several previous studies and offer new insights and treatment approaches for lung stem cell therapy by indicating that club cells and BASCs are crucial for lung damage healing. Researchers then developed a dual homologous recombination system using genetic tools to study the alveolar epithelial repair potential of club cells. By overexpressing the p21 protein, they inhibited the proliferation of AT2 cells and BASCs, revealing club cells as the primary source of alveolar stem cell regeneration. Club‐originated alveolar epithelial cells can cover most of the alveolar area in severely damaged lobes, highlighting the potential of club‐originated alveolar epithelial cells.

**FIGURE 1:**
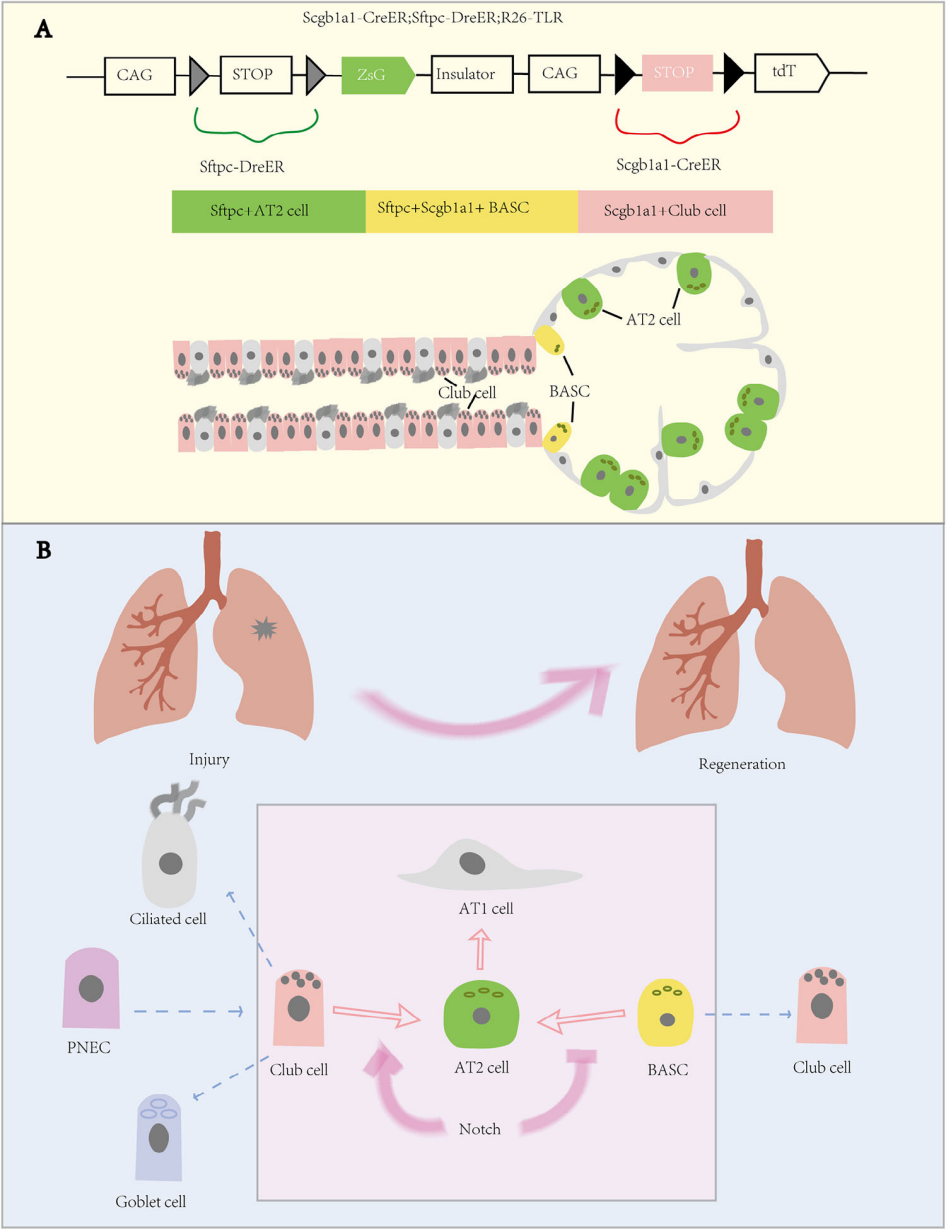
Schematic representation of the new dual homologous recombinase‐mediated genetic profiling tracer technique. (A) The new dual homologous recombination tool called Scgb1a1‐CreER, Sftpc‐DreER, and R26‐TLR is based on Cre‐loxP and Dre‐rox. In this approach, distinct fluorescent markers can be expressed by club cells, alveolar type Ⅱ (AT2) cells, and bronchioalveolar stem cells (BASCs), respectively, which allows for the simultaneous specific labeling of these cells. (B) The newborn AT2 cells, in addition to self‐renewal, also originate from club cells and BASCs, but not AT1 cells after lung injury. The transdifferentiation of club cells and BASCs into AT2 cells was discovered to be regulated by the Notch signaling system, which plays a different function in the transdifferentiation of these two cell types.

Researchers have also discovered that the Notch signaling pathway is down‐regulated during the process of club cells and BASCs transdifferentiating into AT2 cells, suggesting that the Notch signaling system may be involved in the regulation of club cells and BASCs to AT2 cells. One of the most common intercellular signaling pathways, the Notch signaling system is crucial for many biological processes, such as cell proliferation, differentiation, and death.^5^ The authors used conditional gene knockdown and double homologous recombination profiling tracer technologies to knock down the Notch signaling pathway in club cells and BASCs. This allowed them to better understand the role of the Notch signaling pathway in the transdifferentiation of AT2 cells. The Notch knockdown group showed a significant reduction in the proportion of AT2 cells transdifferentiated from club cells, while the proportion of AT2 cells transdifferentiated from BASCs increased. Overexpression of Notch also prevented the transdifferentiation of BASCs to AT2 cells. These findings imply that club cells can act as the AT2 cell's regenerative source and that the Notch pathway's opposing regulation of club cells and BASCs occurs during the latter cell's development into AT2 cells (Figure 1B).

Even while this study only looks at models of lung injury brought on by bleomycin, hyperoxia, or pneumonectomy, it is impossible to rule out the possibility that AT1 could turn into AT2 in models of lung injury caused by other factors. For example, a study by Edward E. Morrisey et al. published in *cell* in 2023 found that actin remodeling and cytoskeletal strain mediated by Cdc42 and Ptk2 preserve AT1 cell destiny at homeostasis and AT1 cells will be reprogrammed to AT2 cells in Cdc42 or Ptk2 knockout mouse models.^6^ Without doubt, more research will be needed to clarify the process by which club cells and BASCs differentiate into AT2 cells during aberrant healing and remodeling in patients with acute or chronic lung tissue injury. In this study, by constructing a precise dual homologous recombination lineage tracer technology to specifically label different lung epithelial cells, the author accurately resolved the regenerative origins of alveolar epithelial stem cells after lung injury. With the use of this novel technique, combined with techniques such as single‐cell sequencing, scientists may be able to better understand the cellular and molecular processes behind lung regeneration, which may result in the identification of novel therapeutic targets and the development of fresh treatment approaches. In addition, previous studies show differences between mouse and human lung regeneration, but these findings are limited to mouse lung injury regeneration, necessitating further research on human lung injury and repair mechanisms.^5^

To sum up, the researchers found, in an innovative way, that the newborn AT2 cells, in addition to self‐renewal, also originate from club cells and BASCs, but not AT1 cells. The Notch signaling pathway, which has a distinct role in the transdifferentiation of these two cell types, regulated the transdifferentiation of club cells and BASCs into AT2 cells. This research offers fresh approaches to the study of lung stem cells, repair, and regeneration. It also identifies novel targets for therapeutic intervention and establishes a solid foundation for future research on lung diseases.

## AUTHOR CONTRIBUTIONS

Z.X. and M.W. provided the conception, funding support, revision, and supervision. Y.W. conducted the literature research, wrote the initial manuscript, and drew the figure. All authors have read and approved to publish the article.

## CONFLICT OF INTEREST STATEMENT

The authors declare no conflict of interest.

## ETHICS STATEMENT

Not applicable.

## Data Availability

Not applicable.

## References

[1] Liu K , Meng X , Liu Z , et al. Tracing the origin of alveolar stem cells in lung repair and regeneration. Cell. 2024;187(10):2428‐2445. 38579712 10.1016/j.cell.2024.03.010

[2] Desai TJ , Brownfield DG , Krasnow MA . Alveolar progenitor and stem cells in lung development, renewal, and cancer. Nature. 2014;507(7491):190‐194. 24499815 10.1038/nature12930 PMC4013278

[3] Sauer B , Henderson N . Site‐specific DNA recombination in mammalian cells by the Cre recombinase of bacteriophage P1. Proc Natl Acad Sci U S A. 1988;85(14):5166‐5170. 2839833 10.1073/pnas.85.14.5166 PMC281709

[4] Kathiriya JJ , Brumwell AN , Jackson JR , Tang X , Chapman HA . Distinct airway epithelial stem cells hide among club cells but mobilize to promote alveolar regeneration. Cell Stem Cell. 2020;26(3):346‐358. 31978363 10.1016/j.stem.2019.12.014 PMC7233183

[5] Basil MC , Cardenas‐Diaz FL , Kathiriya JJ , et al. Human distal airways contain a multipotent secretory cell that can regenerate alveoli. Nature. 2022;604(7904):120‐126. 35355013 10.1038/s41586-022-04552-0 PMC9297319

[6] Shiraishi K , Shah PP , Morley MP , et al. Biophysical forces mediated by respiration maintain lung alveolar epithelial cell fate. Cell. 2023;186(7):1478‐1492 e15. 36870331 10.1016/j.cell.2023.02.010 PMC10065960

